# Inhibition of Glutaredoxin 5 predisposes Cisplatin-resistant Head and Neck Cancer Cells to Ferroptosis

**DOI:** 10.7150/thno.46903

**Published:** 2020-06-19

**Authors:** Jaewang Lee, Ji Hyeon You, Daiha Shin, Jong-Lyel Roh

**Affiliations:** 1 Department of Otorhinolaryngology-Head and Neck Surgery, CHA Bundang Medical Center, CHA University School of Medicine, Seongnam, Republic of Korea.; 2 Western Seoul Center, Korea Basic Science Institute, Seoul, Republic of Korea.

**Keywords:** Ferroptosis, glutaredoxin 5, cancer cells, gene silencing, free iron

## Abstract

**Rationale:** Loss of iron-sulfur cluster function predisposes cancer cells to ferroptosis by upregulating iron-starvation response, but the role of glutaredoxin 5 (GLRX5) silencing in ferroptosis remains unknown. We examined the role of GLRX5 functional loss in promoting ferroptosis in cisplatin-resistant head and neck cancer (HNC) cells.

**Methods:** The effects of sulfasalazine treatment and GLRX5 gene silencing were tested on HNC cell lines and mouse tumor xenograft models. These effects were analyzed concerning cell viability and death, lipid reactive oxygen species (ROS) and mitochondrial iron production, labile iron pool, mRNA/protein expression, and malondialdehyde assays.

**Results:** Cyst(e)ine deprivation, erastin, or sulfasalazine induced ferroptosis in HNC cells, which was relatively less sensitive in cisplatin-resistant HNC cells. Sulfasalazine or cyst(e)ine deprivation-induced ferroptosis resulted from increased lipid peroxidation and intracellular free iron, which were significantly promoted by short-interfering RNA or short hairpin RNA (shRNA) targeting GLRX5 (*P*<0.05). GLRX5 silencing activated iron-starvation response and boosted up intracellular free iron through the iron-responsive element-binding activity of increased iron regulatory protein (increased transferrin receptor and decreased ferritin). These effects were rescued by resistant GLRX5 cDNA but not by catalytically inactive mutant GLRX5 K101Q. The same results were noted in an *in vivo* mouse model transplanted with vector or shGLRX5-transduced HNC cells and treated with sulfasalazine.

**Conclusion:** Our data suggest that inhibition of GLRX5 predisposes therapy-resistant HNC cells to ferroptosis.

## Introduction

Iron is an essential nutrient that is necessary for life but can also cause cell death. Growing epidemiological evidence has shown that excess iron is associated with increased cancer incidence and risk 1. The acquisition and retention of excess iron also lead to increased tumor proliferation, growth, metastasis, and an aggressive phenotype contributing to therapeutic resistance 2. Iron dependence of cancer cells might be a novel therapeutic target via inducing non-apoptotic iron-dependent cell death (ferroptosis) 3. Ferrous iron can form cytotoxic lipid radicals by reacting with lipid peroxides, causing that selectively kill cancer cells, particularly in a therapy-resistant mesenchymal state to evade annihilation 4. Resistant cancer cells exhibit an exquisite vulnerability to the inhibition of glutathione peroxidase 4 (GPX4), an essential regulator of ferroptosis, which potentially represents a novel therapeutic strategy for fighting resilient cancers with iron 4, 5.

Ferroptosis can be regulated by alterations in iron metabolism and a network of iron-dependent proteins. CDGSH iron-sulfur domain (CISD) 1 or 2 protects against mitochondrial injury in ferroptosis by stabilizing the iron-sulfur cluster (ISC) and inhibiting mitochondrial iron uptake and lipid peroxidation 6, 7. Inhibition of CISD1 or 2 contributes to ferroptosis induced by treatment of erastin or sulfasalazine (SAS), the inhibitors of system x_c_^-^ cyst(e)ine/glutamate antiporter (xCT) 6, 7. Cysteine desulfurase, encoded by the *NFS1* gene, is one of the important iron-sulfur cluster (ISC) cofactors presenting in multiple essential proteins upon a high oxygen environment 8. Inhibition of NFS1 activates iron-starvation response and triggers ferroptosis in combination with inhibition of intracellular cysteine transport 8.

Glutaredoxin 5 (GLRX5) is an essential protein engaging in mitochondrial ISC transfers to iron regulatory protein (IRP), m-aconitase, and ferrochelatase 9. GLRX5 deficiency impairs heme biosynthesis, causing sideroblastic anemia and variant nonketotic hyperglycinemia in humans 10. Mutations of GLRX5 lead to distinct effects on downstream ISC biosynthesis and maturation 11. Genetic inhibition of GLRX5 activates the iron-responsive element (IRE)-binding activity of IRP, responsive to cytosolic iron depletion 10. This may upregulate the iron-starvation response and boost intracellular free iron, the prerequisite condition of lipid peroxidation for ferroptosis. However, the role of GLRX5 silencing in ferroptosis remains unknown. The present study has newly found the therapeutic possibility of GLRX5 inhibition predisposing therapy-resistant cancer cells to ferroptosis. Here, we examined the role of GLRX5 functional loss in promoting ferroptosis in cisplatin-resistant head and neck cancer (HNC) cells.

## Materials and Methods

### Cell culture

Head and neck squamous cell carcinoma cell lines, namely AMC-HN3, HN3R, HN4, and HN4R 12, were used for our experiments. HN3R and HN4R were the cisplatin-resistant cell lines developed from the parental cisplatin-sensitive cell lines of HN3 and HN4, respectively 13, 14. These cell lines were authenticated by short tandem repeat-based DNA fingerprinting and multiplex polymerases chain reaction (PCR). The cells were cultured in Eagle's minimum essential medium (Sigma-Aldrich, St. Louis, MO, USA) supplemented with 10% fetal bovine serum at 37°C in a humidified atmosphere containing 5% CO_2_. The cells were also cultured in the conditioned media with no cysteine and cystine (MP Biomedicals, Irvine, CA, USA), or with exposure to erastin (Sellechem, Houston, TX, USA) or sulfasalazine (SAS) (Sigma-Aldrich).

### Cell death and viability assays

The cells were exposed to SAS for indicated time and dose, and cell death was assessed via propidium iodide (PI) staining. Control cells were exposed to an equivalent amount of dimethyl sulfoxide (DMSO). The cell death was also measured with or without pretreatment of 100 µM deferoxamine (Abcam, Cambridge, UK), or co-incubation of 2 µM ferrostatin-1 (Sigma-Aldrich) or 200 µM α-tocopherol (Sigma-Aldrich). The sample was washed twice with phosphate-buffered saline (PBS), followed by staining of cells in each plate with 2.5 μg/mL PI (Sigma-Aldrich) in PBS for 30 min. The stained cells were analyzed using a FACSCalibur flow cytometer equipped with CellQuest Pro (BD Biosciences, San Jose, CA, USA) and observed using a ZEISS fluorescent microscope (Oberkochen, Germany). The mean PI-positive fractions were compared with those of the control group or between different treatment groups.

Cell viability after exposure to cyst(e)ine deprivation or erastin or SAS treatment was assessed using the tetrazolium compound 3-[4,5-dimethyl-2-thiazolyl]-2,5-diphenyl-2H-tetrazolium bromide (MTT) (Sigma-Aldrich). MTT assays were performed 4 h followed by a solubilization buffer for 2 h and absorbance was then measured at 570 nm using a SpectraMax M2 microplate reader (Molecular Devices, Sunnyvale, CA).

### Measurement of ROS production, lipid peroxidation, and GSH synthesis

Cellular ROS or lipid ROS generation was measured in the HNC cells treated with 1 mM SAS treatment for 8 h, by adding 10 µM 2ʹ,7ʹ-dichlorofluorescein diacetate (DCF-DA) (cellular ROS; Enzo Life Sciences, Farmingdale, NY, USA) or 5 µM BODIPY-C11 (lipid peroxidation; Thermo Fisher Scientific) for 30 min at 37°C. The ROS levels were analyzed using a FACSCalibur flow cytometer equipped with CellQuest Pro (BD Biosciences). Cellular lipid peroxidation was also assessed in HNC cell lysates by measuring the concentration of malondialdehyde (MDA), an end product of lipid peroxidation, using a lipid peroxidation assay kit (Abcam, Cambridge, MA, USA). Intracellular glutathione (GSH) levels in HNC cell lysates subjected to 1 mM SAS treatment for 8 h were measured using a GSH colorimetric detection kit (BioVision Inc., Milpitas, CA, USA).

### Labile iron pool, mitochondrial iron and superoxide generation assays

Labile iron pool (LIP) assay was measured by using calcein acetoxymethyl ester (Corning Inc., Corning, NY, USA) and iron chelator, deferoxamine. The cells were loaded with 2 μM calcein for 30 min at 37 °C and then washed with Hanks' balanced salt solution (HBSS). Deferoxamine was added at a final concentration of 100 µM to remove iron from calcein, causing dequenching. The change in fluorescence following the addition of deferoxamine was used as an indirect measure of the LIP. Fluorescence was measured at 485 nm excitation and 535 nm emissions with a fluorescence plate reader (BioTek, Winooski, VT, USA). Mitochondria were isolated using the mitochondrial isolation kit (Thermo Fisher Scientific). The ferrous iron level in the cell or mitochondria was measured using the iron assay kit (Sigma-Aldrich). The accumulation of intra-mitochondrial iron was also measured with rhodamine B-[(1,10-phenanthroline-5-yl)-aminocarbonyl]benzyl ester (RPA), a fluorescent non-toxic iron sensor (Squarix GmbH, Marl, Germany). Arbitrary fluorescence units (AUs) were compared among differently treated groups. The mean value of each group was normalized to that of the control group.

### RNA interference and gene transfection

For silencing the *GLRX5* gene, HN3R and HN4R cells were seeded. Cells were transfected 24 h later with 10 nmol/L small-interfering RNA (siRNA) targeting human *GLRX5* or scrambled control siRNA (Integrated DNA Technologies, Coralville, IA, USA) using Lipofectame RNAiMAX reagent (Thermo Fisher Scientific). The HN3R, HN4, and HN4R cells were stably transduced with short hairpin RNA (shRNA) targeting *GLRX5* (Integrated DNA Technologies). To generate cells that stably overexpress *GLRX5*, HN4R cells were stably transfected with a control plasmid (pBABE-puro, Addgene, Watertown, MA, USA), a resistant *GLRX5* cDNA (GLRX5res, gBocks® gene fragment GLRX5, Integrated DNA Technologies)-cloned plasmid, or a catalytically inactive mutant *GLRX5* cDNA (GLRX5res K101Q)-cloned plasmid produced using EZchange^TM^ site-directed mutagenesis kit (Ezynomics, Daejeon, Republic of Korea). The GLRX5 mutation (K101Q) was adopted from the previous report of distinct functionalities of GLRX5 mutants that prevent the binding of Fe-S to GLRX5 protein and affect aconitase and α-ketoglutarate dehydrogenase (αKGDH) activities 11. The sequences of the resulting plasmids containing wild type or mutant *GLRX5* were verified by direct sequencing. GLRX5 expression was also confirmed via Western blotting and reverses transcription-quantitative PCR.

### Immunoblotting and reverse transcription-quantitative PCR

Cells were plated and grown with 70% confluence, and then subjected to treatment with SAS. For immunoblotting, cells were lysed at 4°C in a cell lysis buffer (Cell Signaling Technology, Danvers, MA, USA) with protease/phosphatase inhibitor cocktail (Cell Signaling Technology). A total of 5-25 µg protein was resolved by SDS-PAGE on 10%-15% gels; the resolved proteins were then transferred to nitrocellulose or polyvinylidene difluoride membranes and probed with primary and secondary antibodies. The following primary antibodies were used: Grx5 (bs-13395R, Bioss, Melbourne, FL, USA), xCT (ab37185, Abcam, Cambridge, UK), Gpx4 (ab125066, Abcam), IRP 2 (sc-33682, Santa Cruz Biotechnology, Inc., Dallas, TX, USA), transferrin receptor protein (TfR) 1 (NB100-92243, Novus, St. Louis, MO, USA), ferritin heavy chain (FTH1) (4393S, Cell Signaling Technology), and ferroportin (Fpn; NBP1-21502, Novus). β-actin (BS6007M, BioWorld, Atlanta, GA, USA) served as the total loading control. All antibodies were diluted to concentrations between 1:500 and 1:10000.

Total RNA from HNC cells was also extracted using an RNA extraction kit (Genolution, Seoul, Republic of Korea) according to the manufacturer's instructions. A reverse transcription-quantitative PCR from 1-2 µg total RNA for each extracted sample was conducted using SensiFAST™ SYBR^®^ No-ROX Kit (Bioline International, Toronto, Canada) after performing cDNA synthesis using SensiFAST™ cDNA Synthesis Kit (Bioline International). *TFRC, IREB2, SLC40A1*, and *ACTB* were amplified, and the relative target mRNA levels were determined using the 2^-(ΔΔCt)^ method and normalized against *ACTB* mRNA levels and then to the control.

### Assays for analyzing aconitase and αKGDH activities

The aconitase and αKGDH activities were examined in HN4R cells, according to the manufacturer's protocol of an aconitase activity colorimetric assay kit (K716-100, BioVision Inc.) and αKGDH assay kit (K678-100, BioVision Inc.), respectively. HN4R cells were stably transduced with vector control or shGLRX5 and GLRX5res or GLRX5 res (K101Q) and then exposed to 1 mM SAS for 8 h.

### Tumor xenograft

All animal study procedures were performed in accordance with protocols approved by the Institutional Animal Care and Use Committee (IACUC). Five-week-old athymic BALB/c male nude mice (nu/nu) were purchased from Central Lab Animal Inc. (Seoul, Republic of Korea). HN4R cells with vector control or shGLRX5 were subcutaneously injected into the bilateral flank of nude mice. From the day when gross nodules were detected in tumor implants, mice were subjected to different treatments: vehicle or SAS (250 mg/kg daily per intraperitoneal route) 15. Each group included seven mice. Tumor size, body weight, and food intake of each mouse were measured twice a week, and tumor volume was calculated as (length×width^2^)/2. After the scarification of mice, tumors were isolated and analyzed by measuring cellular lipid ROS, iron and MDA contents, and immunoblotting. These were compared among differently treated tumors.

### Statistical analysis

Data were presented as mean±standard error of the mean. The statistically significant differences between the treatment groups were assessed using Mann-Whitney *U*-test or analysis of variance (ANOVA) with Bonferroni post-hoc test. The expression levels of GLRX5 mRNA were obtained from the normal mucosa (*n*=43) and HNC (*n* = 519) datasets of TCGA assessed from the cBioPortal (www.cbioportal.org). The median values of low and high expression levels of GLRX5 mRNA were determined and compared using *t*-test. Univariate Cox proportional hazards regression analyses were used to identify associations between GLRX5 mRNA expression levels and overall survival or disease-free survival in the HNC cohort. The Kaplan-Meier and log-rank tests were used to determine and statistically compare the survival rates, respectively. All statistical tests were two-sided and a *P* value of <0.05 was considered to be statistically significant. The statistical tests were performed using IBM SPSS Statistics version 22.0 (IBM, Armonk, NY, USA).

## Results

### Inhibition of GLRX5 promotes ferroptotic cell death of HNC cells

**Figure 1:**
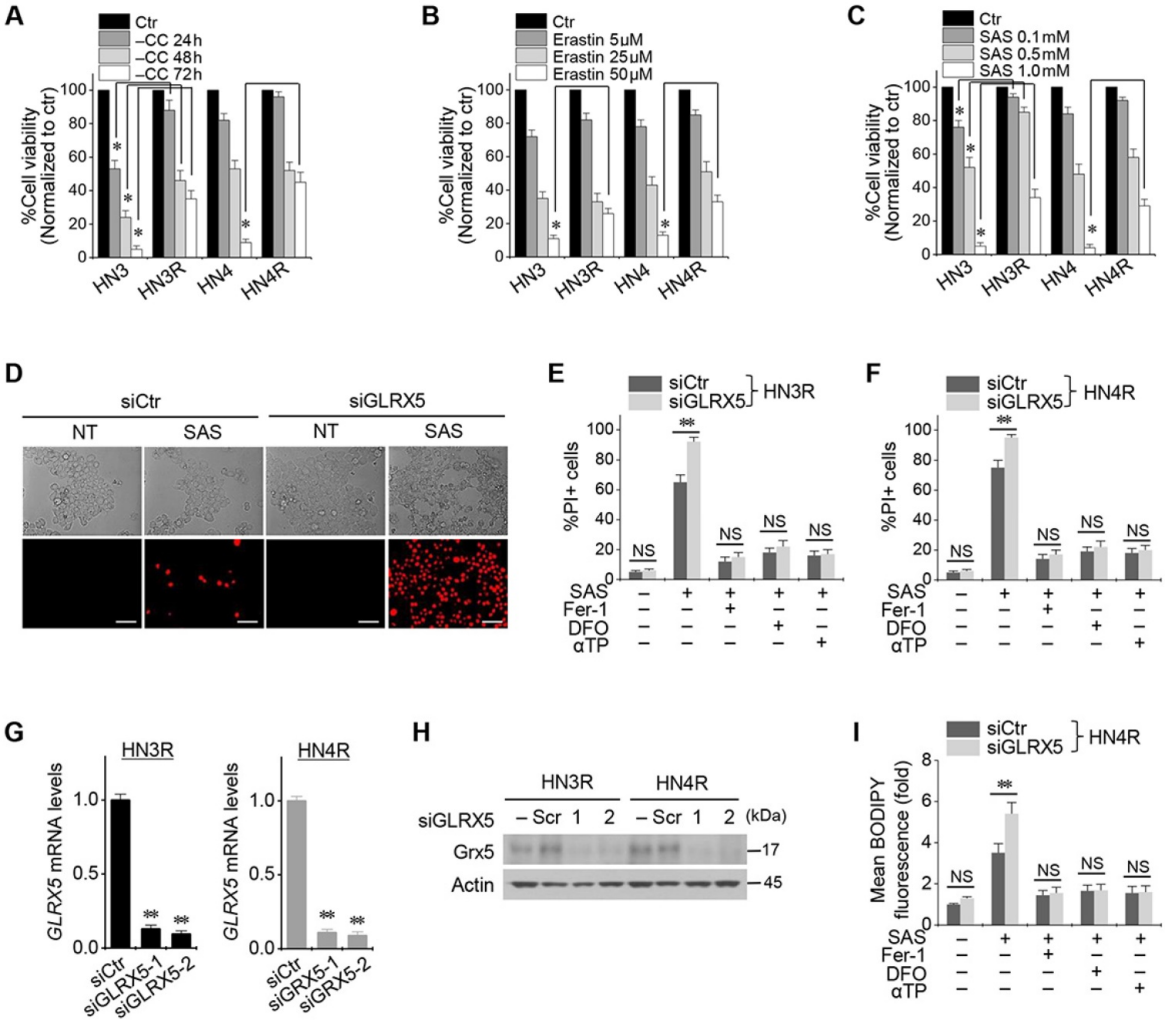
Inhibition of GLRX5 promotes ferroptotic cell death of head and neck cancer (HNC) cells. (***A***-***C***) Cell viability of HNC cells exposed to cyst(e)ine (CC) deprivation and erastin or sulfasalazine (SAS) treatment in HNC cells. The cell viability was relative to control not treated with CC deprivation, erastin, or SAS. HN3R and HN4R were the cisplatin-resistant HNC cell lines developed from the parental cisplatin-sensitive cell lines of HN3 and HN4, respectively. * *P*<0.05. (***D***-***F***) Ferroptosis in HNC cells induced by SAS treatment. Propidium iodide (PI)-positive cells were stained and counted using fluorescent microscopy and flow cytometry after 1 mM SAS treatment for 48 h. NT indicates control treated with DMSO only. Scale bar, 50 μm. ** *P* < 0.01. NS indicates statistically not significant. (***G***, ***H***) mRNA and protein expression of GLRX5 in the HN3R and HN4R cells transfected with siRNA control (siCtr) or siGLRX5. (***I***) Changes of cellular lipid reactive oxygen species (ROS) levels of HN4R cells exposed to 1 mM SAS for 8 h. The cells were also pretreated with deferoxamine (DFO, 100 μM) or co-treated with ferrostatin-1 (Fer-1, 2 μM), or α-tocopherol (αTP, 200 μM). The error bars represent standard errors from three replicates. ** *P* < 0.01 between siCtr and siGLRX5.

Cyst(e)ine deprivation, erastin, or SAS induced ferroptosis in HNC cell lines. The viabilities of HN3 and HN4 cells decreased in a time-dependent manner of cyst(e)ine deprivation and a dose-dependent manner of erastin or SAS (Figure 1A-C). Cisplatin-resistant HN3R and HN4R cell lines were relatively less sensitive to cyst(e)ine deprivation or erastin or SAS treatment compared with HN3 and HN4 cells (*P* < 0.05). Further experiments were performed to verify whether the less sensitivity of ferroptosis inducers in the cisplatin-resistant HNC cells was overcome by the inhibition of GLRX5. Genetic silencing of GLRX5 considerably increased the PI-positive cell fractions of HN3R and HN4R by SAS-induced ferroptosis (*P* < 0.01) (Figure 1D-F). The cell death was pharmacologically inhibited when pretreated with deferoxamine or co-treated with ferrostatin-1 or α-tocopherol. When siGLRX5 was transfected in both HNC cell lines, the mRNA and protein expression of GLRX5 significantly decreased (*P*< 0.001) (Figure 1G, 1H). SAS is known to induce ferroptosis by cellular lipid peroxidation and GSH depletion from targeting cyst(e)ine/glutamate antiporter system X_c_^-^ 16 and therefore, was treated in the both cisplatin-sensitive and -resistant HNC cells. GLRX5 gene silencing also induced significantly increased lipid ROS production in the SAS-treated cancer cells compared with vector control (*P* < 0.01) (Figs. 1G-I).

### GLRX5 genetic silencing enhances ferroptosis, lipid peroxidation, and free iron accumulation

**Figure 2:**
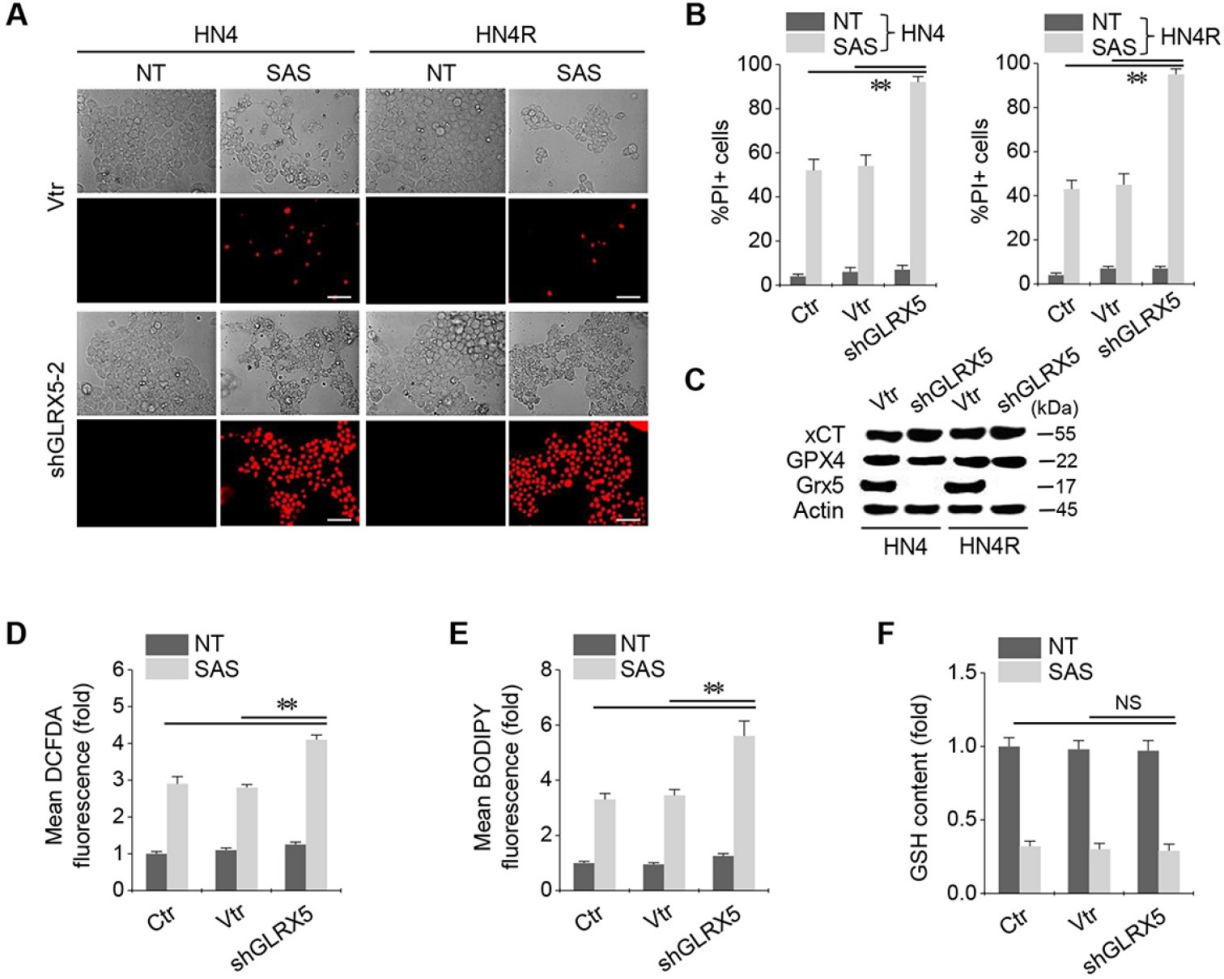
GLRX5 genetic silencing increases ferroptosis and lipid peroxidation. (***A*-*C***) Cell death and protein expression in HN4 and HN4R cells stably transduced with shRNA targeting GLRX5 or vector control (vtr). The cells were subjected to 0.5 mM SAS for 48 h. Scale bar, 50 μm. ** *P* < 0.01 relative to a non-vector control (ctr) or vtr. (***D***-***F***) Cellular ROS, lipid ROS (BODIPY), and glutathione (GSH) contents in HN4R cell with or without GLRX5 gene silencing, which were subjected to 1 mM SAS treatment for 8 h. The error bars represent standard errors from three replicates. ** *P* < 0.01 relative to ctr or vtr. NS indicates statistically not significant.

When shGLRX5 or a vector was stably transduced in the HN4 and HN4R cells, PI-positive cell fractions significantly increased in both cell lines with SAS treatment (*P* < 0.01) (Figure 2A-B). The stable transduction of shGLRX5 significantly decreased the protein expression of GLRX5 (*P*< 0.001) but none of xCT or GPX4, two key molecules related to ferroptosis (Figure 2C). SAS induced a significant increase in total and lipid ROS levels (measured by DCFDA and BODIPY-C11, respectively) and a decrease in GSH contents in HNC cells (*P* < 0.01). Along with GLRX5 gene silencing, cellular and lipid ROS significantly increased compared to a non-vector or vector control (*P* < 0.01) (Figure 2D-E). GSH levels decreased by SAS treatment but the changes of GSH contents did not significantly differ between the controls and shGLRX5 gene-silenced HN4R cells (*P* > 0.1) (Figure 2F).

**Figure 3:**
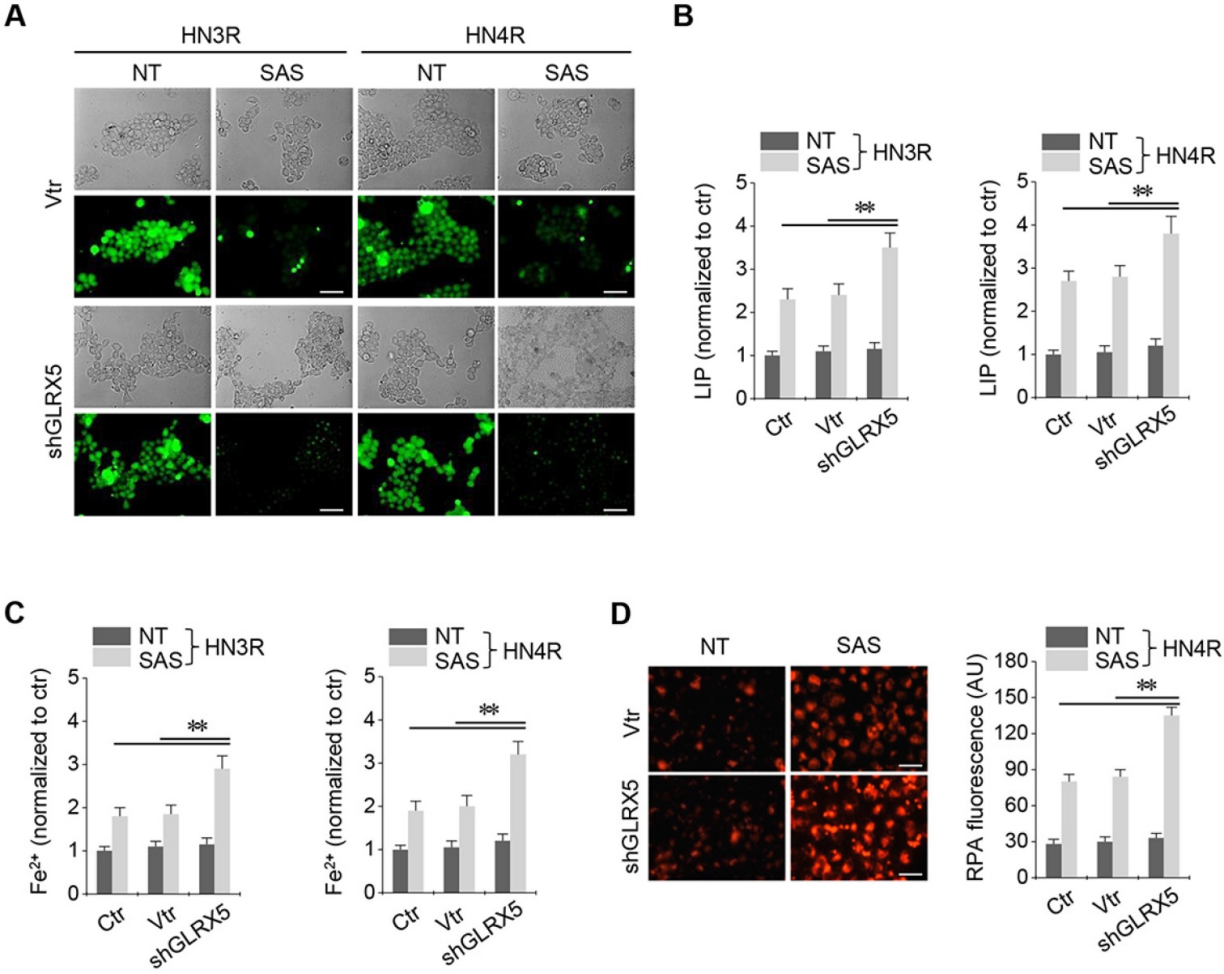
Inhibition of GLRX5 elevates intracellular free iron levels. (***A***, ***B***) Labile iron pool (LIP) in HN3R and HN4R cells with or without shGLRX5 gene silencing. LIP assay was measured in the HNC cells that were treated with or without 1 mM SAS for 8 h. (***C***) Ferrous iron (Fe^2+^) assay in both HNC cell lines. (***D***) mitochondrial iron accumulation in HN4R cells, which were subjected to 1 mM SAS treatment for 8 h. Mitochondrial iron was measured using rhodamine B-[(1,10-phenanthroline-5-yl)-aminocarbonyl]benzyl ester (RPA). The error bars represent standard errors from three replicates. Scale bar, 50 μm. ** *P* < 0.01 relative to ctr or vtr.

Stable transduction of GRX shRNA and vector was established in the cisplatin-resistant HN3R and HN4R cells. SAS treatment in the HNC cells induced a significant increase in labile iron and ferrous iron (Fe^2+^) levels. LIP increased by SAS treatment, which was significantly higher in the shGLRX5-transduced cancer cells than in the non-vector and vector controls (*P* < 0.01) (Figure 3A-B). Cellular and mitochondrial Fe^2+^ levels after SAS treatment were significantly higher in the shGLRX5-transduced cancer cells than the controls (*P* < 0.01) (Figure 3C-D).

The same findings were examined in the HNC cells cultured in a condition of cyst(e)ine deprivation. Cyst(e)ine deprivation induced ferroptosis of HNC cells similar to that observed in SAS-treated cells. Inhibition of *GLRX5* gene also enhanced ferroptotic cell death, increased cellular ROS levels and lipid peroxidation, and free iron accumulation (Figure S1-2).

### Resistant GLRX5 cDNA restores increased ferroptosis and free iron levels

**Figure 4:**
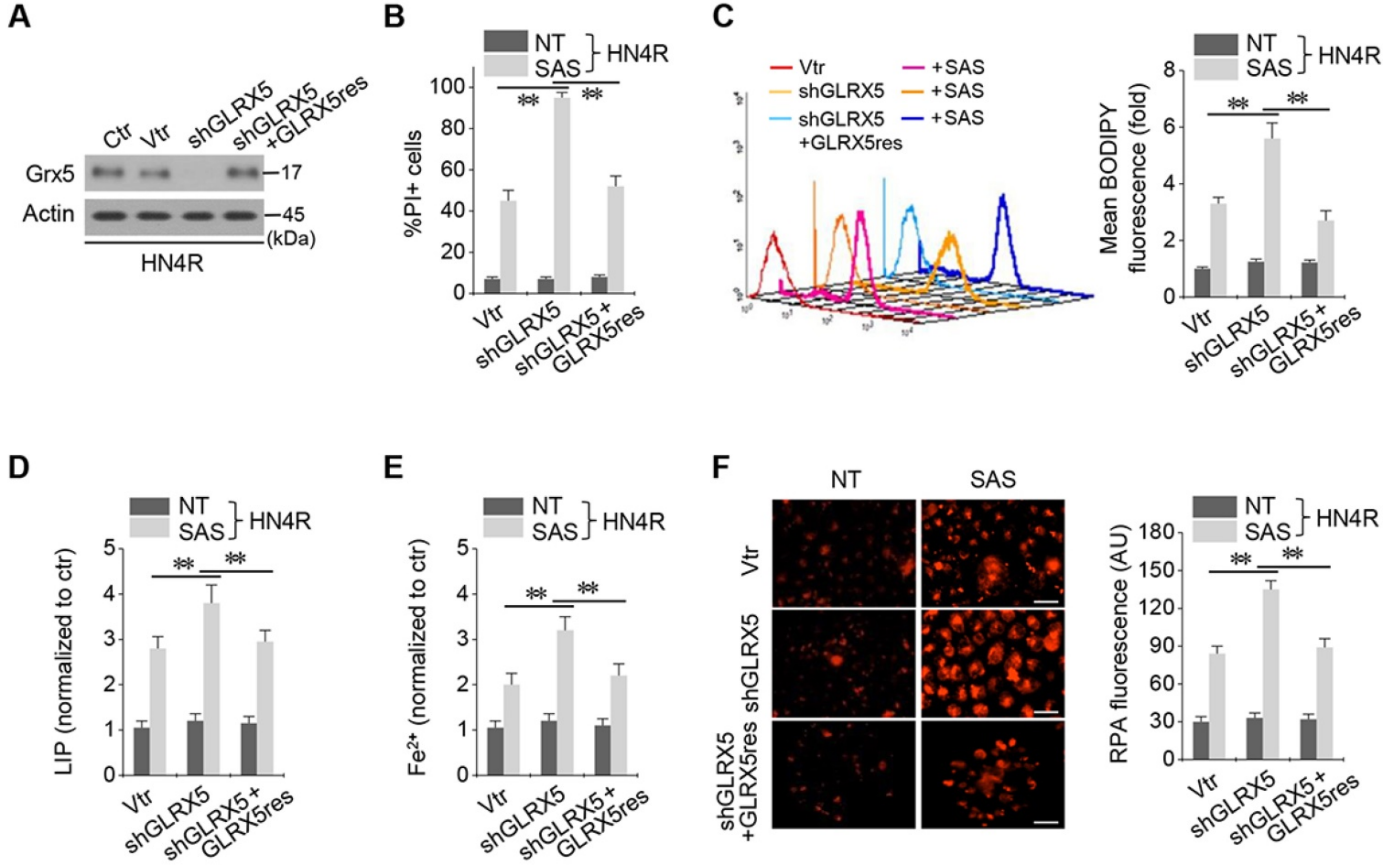
Resistant GLRX5 cDNA transduction rescues ferroptosis and free iron increases. (***A***-***C***) Immunoblots, cell death, and lipid ROS in HN4R cells stably transduced with shRNA targeting a vector or GLRX5 and resistant GLRX5 cDNA (GLRX5res). The cells were exposed to 0.5 mM SAS for 48 h (PI+ cells) or 1 mM SAS 8 h (BODIPY). (***D***-***F***) LIP, Fe^2+^, and mitochondrial iron assays in HN4R cells stably transduced with a vector or shGLRX5 and GLRX5res, which were exposed to 1 mM SAS treatment for 8 h. The error bars represent standard errors from three replicates. Scale bar, 50 μm. ** *P* < 0.01 between the different groups treated with SAS.

The cisplatin-resistant HN4R cells were stably transduced with shRNA targeting GLRX5 with or without co-transduction of a resistant *GLRX5* cDNA (GLRX5res) or a catalytically inactive mutant *GLRX5* cDNA (GLRX5res K101Q). GLRX5res transduction restored the protein expression of GLRX5 suppressed by shGLRX5 stable transduction in HN4R cells (Figure 4A). PI-positive cell fraction by SAS treatment more increased in the shGLRX5-transduced cells than a vector control, which restored to the level of the vector control in HN4R cells transduced by shGLRX5 and GLRX5res (Figure 4B). Lipid ROS, LIP, free iron, and mitochondrial iron accumulation after SAS treatment significantly increased by shGLRX5 gene silencing, which was restored by the GLRX5res transduction (*P* <0.01) (Figure 4C-F). The same findings were also observed in the cisplatin-resistant HN4R cells with transduction of vector, shGLRX5, or shGLRX5 plus GLRX5res when cultured in cyst(e)ine deprivation media (Figure S2).

### Inhibition of GLRX5 activates an iron-starvation response

**Figure 5:**
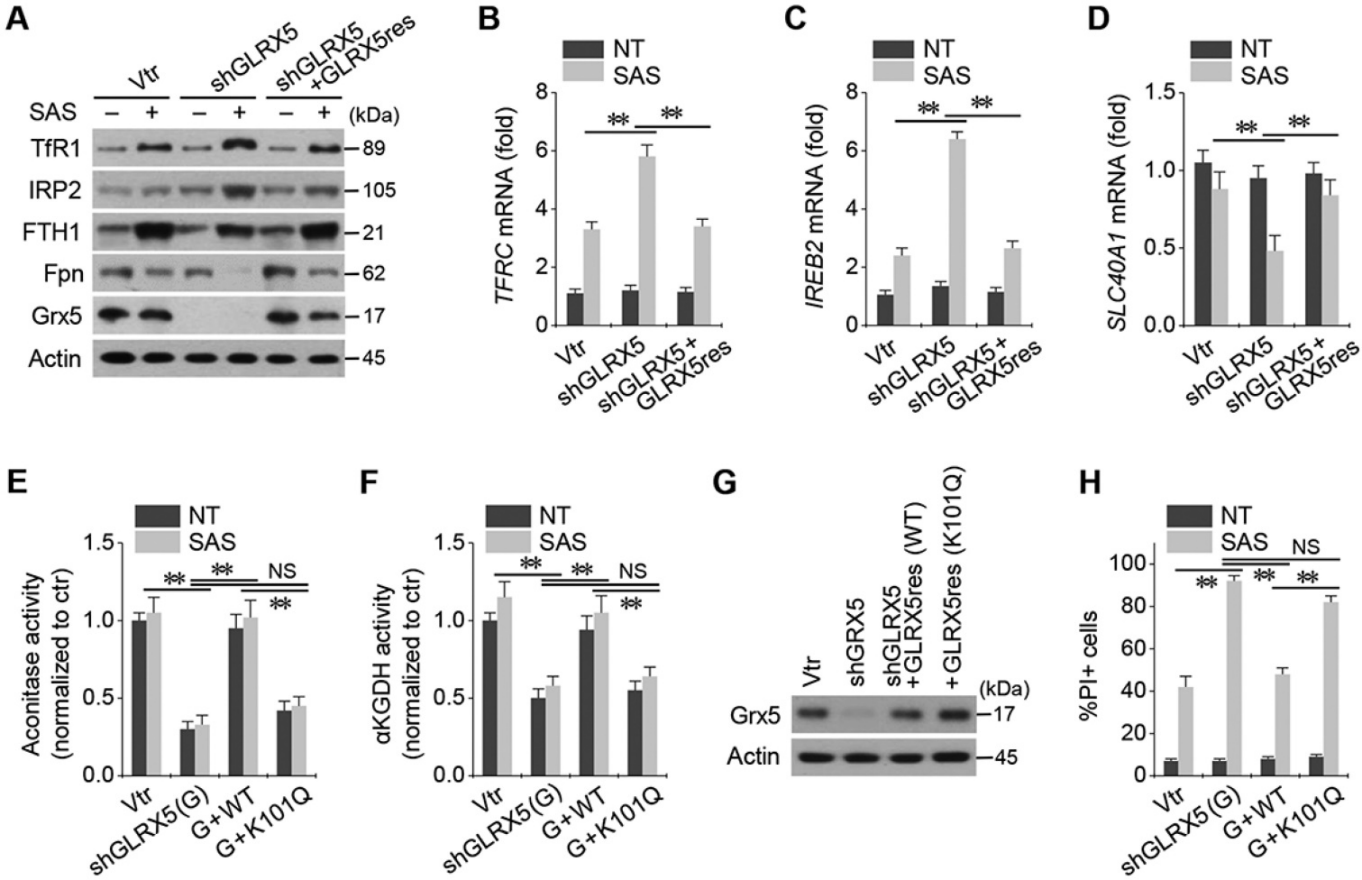
Inhibition of GLRX5 upregulates an iron-starvation response. (***A***-***D***) Protein and mRNA expression of (TfR1, *TFRC*), iron regulatory protein (IRP2, *IREB2*), ferritin (FTH1) and ferroportin (Fpn, *SLC40A1*) genes in HN4R cells stably transduced with a vector or shGLRX5 and GLRX5res, which were subjected to 1 mM SAS treatment for 24 h. (***E***-***H***) Aconitase and α-ketoglutarate dehydrogenase (αKGDH) activities, immunoblots, and cell death of HN4R cells stably transduced with a vector control or shGLRX5 and resistant GLRX5 cDNA (GLRX5res) wild type (WT) or catalytically inactive mutant cDNA (K101Q). The error bars represent standard errors from three replicates. ** *P* < 0.01 between the different groups treated with SAS.

Stable transduction of shGLRX5 and a resistant GLRX5res cDNA or a catalytically inactive mutant GLRX5res K101Q cDNA was established in HN4R cells. GLRX5 genetic silencing boosted up iron-starvation response to SAS treatment. After SAS treatment in HN4R cells stably transduced with shGLRX5, protein and mRNA expressions of TfR1 and IRP2 increased but those of FTH1 and Fpn decreased, which were restored by co-transduction of resistant GLRX5res (Figure 5A-D). Aconitase and αKGDH activities were also examined because GLRX5 is known to engage in mitochondrial ISC transfers to m-aconitase as well as iron regulatory protein (IRP) and ferrochelatase 9. Aconitase and αKGDH activities significantly decreased by GLRX5 gene silencing (*P* <0.01), which rescued by GLRX5res transduction but not by GLRX5res K101Q (Figure 5E-F). Protein expression of GLRX5 was inhibited by shGLRX5 transduction, which was restored by GLRX5res or GLRX5res K101Q (Figure 5G). PI-positive cell fraction increased by GLRX5 genetic silencing in addition to SAS treatment were also rescued by GLRX5res but not by GLRX5res K101Q (Figure 5H).

### Inhibition of GLRX5 sensitizes HNC cells to SAS treatment *in vivo*

**Figure 6:**
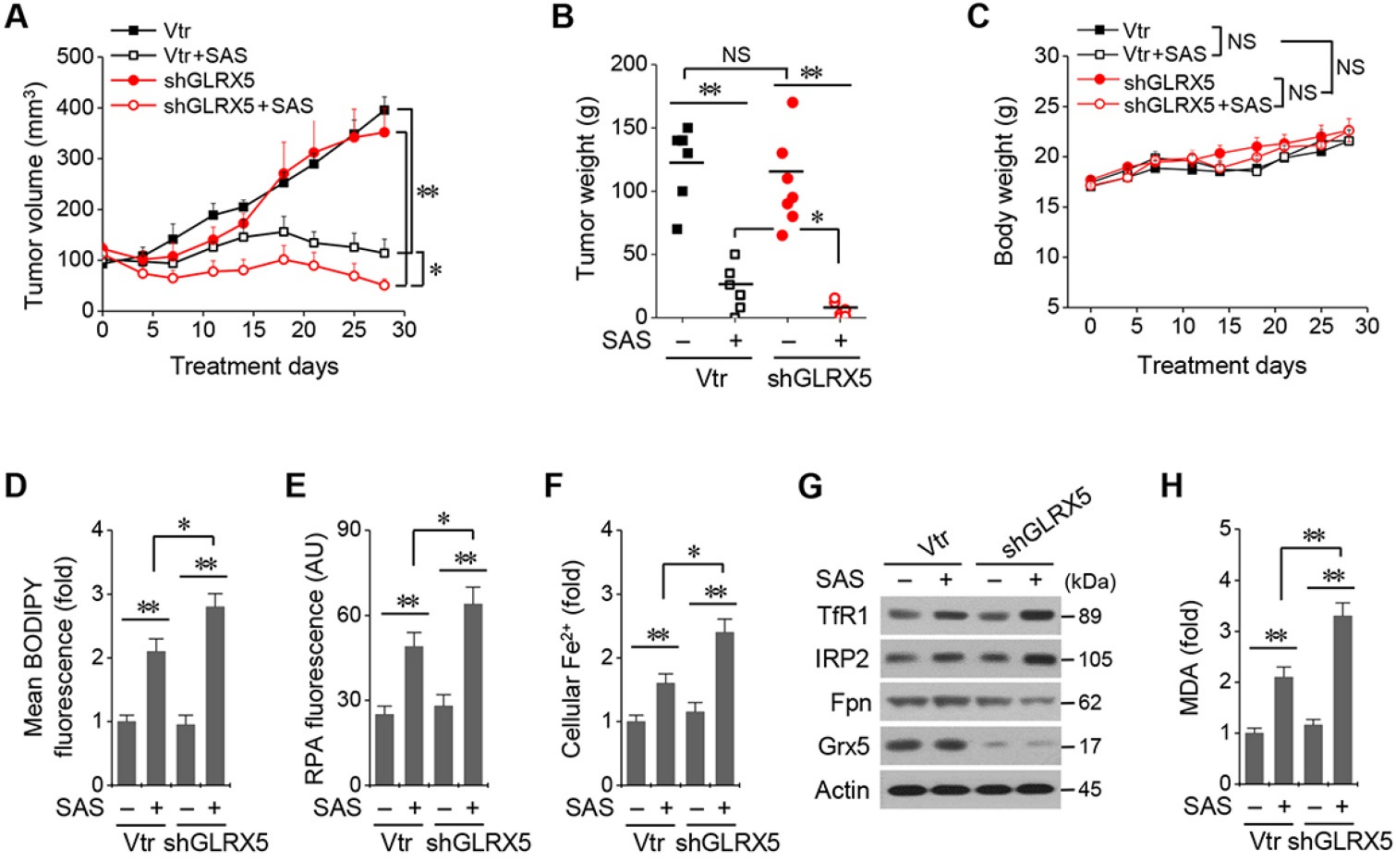
Genetic inhibition of GLRX5 sensitizes therapy-resistant HNC cells to SAS treatment *in vivo*. (***A***-***C***) Tumor growth and weight, and change of body weight and daily food intake after the transplantation of HN4R in nude mice. Tumor volumes were regularly measured after SAS or vehicle treatment in nude mice transplanted with vector or shGLRX5-transduced HN4R cells. (***D***-***H***) Measurements of lipid ROS, RPA, ferrous iron, protein expression, and malondialdehyde (MDA) in tumor tissues with different treatments. The error bars represent standard errors. * *P* <0.05, ** *P* < 0.01 relative to control or other treatment groups.

All the mice survived well during and after tumor cell implantation and treatment with SAS or vehicle. They were euthanized 28 days after treatment. Tumor volume and weights did not differ between cancer cells transduced with the vector and shGLRX5 (Figure 6A-B). SAS treatment significantly suppressed *in vivo* tumor growth compared with the vehicle control (*P* < 0.01), which was more prominent in the mice transplanted with shGLRX5-transduced cancer cells than with vector-transduced cells (*P* < 0.05). Bodyweight (Figure 6C) and daily food intake (not presented) did not change significantly in the control or the SAS treatment group (*P* > 0.05). The levels of lipid ROS, RPA, and ferrous iron were significantly higher in tumor tissues treated by SAS than those of the vehicle control (*P* < 0.01) (Figure 6D-F). Further, the increases of lipid ROS, RPA, and ferrous iron levels by SAS treatment were much higher in the shGLRX5-transduced tumors than the vector-transduced tumors (*P* < 0.05). Representative images of protein expression in tumor tissues showed the accordance with the *in vitro* results (Figure 5A-D) of iron-starvation response boosted up by the GLRX5 silencing (increased TfR1, decreased Fpn) (Figure 6G). The end product of lipid ROS, MDA concentrations in tumor tissues treated by SAS were more increased when tumors experienced GLRX5 silencing (Figure 6H).

### GLRX5 mRNA expression level in and survival of HNC cells

From the TCGA datasets, it was found that mean (±standard deviation) expression levels of GLRX5 did not significantly differ between the HNC samples and normal ones (9.79±0.53 vs. 9.66±0.51, respectively; *P* = 0.097). Median values of GLRX5 mRNA expression were 9.79 (9.45-10.10) in 519 HNC samples and 9.48 (9.29-9.84) in 43 normal samples (Figure S3A). High and low expression levels of GRX mRNA in HNC samples were determined by the median values in the HNC cohort from the TCGA datasets. Univariate Cox proportional hazard regression analysis showed that the expression level of GLRX5 mRNA was not significantly associated with overall survival or disease-free survival outcomes (*P*>0.3) (Figure S3B-C).

## Discussion

**Figure 7:**
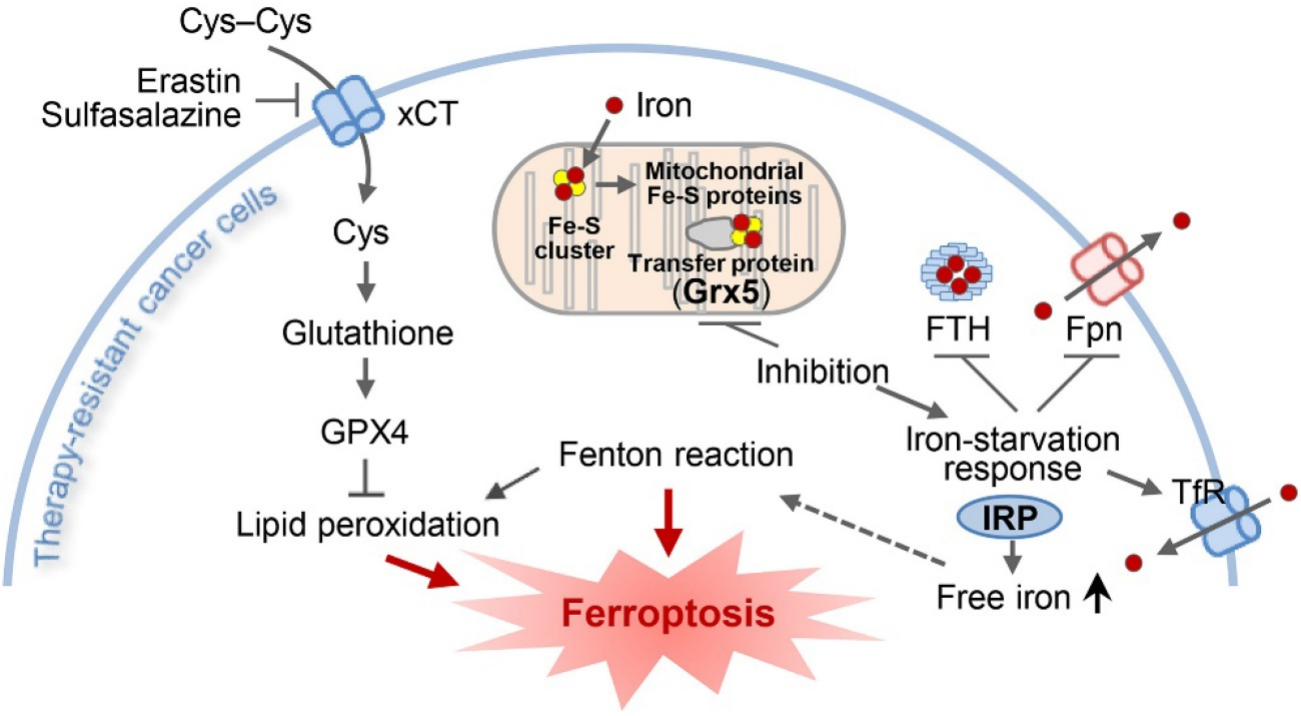
An illustration showing the role of GLRX5 suppression in predisposing therapy-resistant cancer cells to ferroptosis. Erastin or SAS induces ferroptosis by inhibiting the system x_c_^-^ cyst(e)ine/glutamate antiporter (xCT) and depleting glutathione. Inhibition of Grx5, a transfer protein of iron (Fe)-sulfur (S) cluster, upregulates iron-starvation response and increases lipid peroxidation. Increased iron regulatory protein (IRP) and transferrin receptor (TfR), and decreased ferroportin (Fpn) and ferritin (FTH) induce increased intracellular free iron, resulting in promoting ferroptosis in cancer cells by increasing lipid peroxidation.

The present study showed the role of GLRX5 functional loss in promoting ferroptosis in therapy-resistant cancer cells. Cyst(e)ine deprivation, erastin, or SAS induced ferroptosis by depleting GSH and increased lipid peroxidation. Suppression of GLRX5 activated iron-starvation response and increased lipid peroxidation (Figure 7). Increased IRP and TfR and decreased Fpn and FTH boosted up intracellular free iron, resulting in lipid peroxidation and ferroptosis *in vitro* and *in vivo*. This was rescued by GLRX5res but not by mutant GLRX5res K101Q. Therefore, our study showed a new therapeutic potentiality of GLRX5 inhibition predisposing therapy-resistant cancer cells to ferroptosis.

GLRX5 is a mitochondrial protein that plays an essential role in the efficient transfer of ISC trafficked from mitochondrial ISC assembly enzyme ISCU1 9. GLRX5 is engaging in transferring mitochondrial ISC to IRP1, m-aconitase, and ferrochelatase, the proteins essential for cellular iron homeostasis maintenance and heme biosynthesis 10. Deficiency of GLRX5 impacts on downstream ISC proteins, by cytosolic iron depletion and heme biosynthesis inhibition, as observed in the sideroblastic anemia patients and animal models 10, 17. K101Q mutation of GLRX5 significantly reduces aconitase, pyruvate dehydrogenase, and αKGDH activities 11. The present study also showed that GLRX5 genetic silencing or K101Q mutation was associated with the reduced activities of aconitase and αKGDH. Further, the suppression of GLRX5 activated the IRE-binding activity of IRP and canonical iron-starvation responsive proteins (increased TfR, decreased FTH), resulting in increased intracellular free iron.

Iron is imported into cells by binding to TfR1, trafficked in an endosome, stored in ferritin, and exported by Fpn under tightly regulated cellular iron homeostasis at the post-transcriptional level by a network of iron-dependent proteins 18. IRPs are the main components of this intracellular iron network, binding to the IREs that regulate whole processes of the iron import, storage, and export 19. Canonical iron-starvation response stabilizes iron import mRNAs (*TFRC*, *DMT1*) and represses iron storage (*FTH1*, *FTL*) and export (*FPN*) mRNAs 20. Iron-starvation response dramatically increases intracellular free iron or LIP, the source of Fenton chemistry reacting iron-mediated lipid peroxidation. The process is mitigated by GPX4 and the inactivation of GPX4 leads to an accumulation of lipid peroxides 21. As GSH is a cofactor of selenium-dependent GPX4, depletion of intracellular GSH by erastin or SAS indirectly inactivates GPX4 and suffices to induce ferroptosis 22. The cell death can be modulated by the cellular levels of iron reacting with lipid peroxides 23. In the present study, ferroptosis was promoted by increased cellular free iron by following the suppression of GLRX5.

ISC proteins, hemeproteins, and other iron-containing proteins, utilize LIP defined as a low-molecular-weight pool of cellular iron; a lack of these proteins impact on cellular iron homeostasis and regulatory response 24. NFS1 is a gene encoding critical step of ISC biosynthesis that supplies inorganic sulfur to the ISC by removing sulfur from cysteine 25. Suppression of NFS1 activates canonical iron-starvation responsive proteins regardless of the environmental oxygen level and resulted in the same condition as that of iron overloaded cancer cells undergoing ferroptosis 8. Similar results were observed in the present study showing the association between GLRX5 inhibition and iron-starvation response activation resulting in promoting ferroptosis. Therefore, our and previous results might suggest that inhibition of ISC biosynthesis promotes induction of ferroptosis by tricking cancer cells into taking up large quantities of iron and releasing intracellular iron stores that responded to the iron-starvation response 8.

Head and neck squamous cell carcinoma is the most common pathology of HNC arising in the epithelium of the upper aerodigestive tract. Epithelial cancers are relatively less dependent on lipid peroxidase pathway (GPX4) and less sensitive to ferroptosis inducers than cancers in a mesenchymal state 4. Expression of epithelial markers related to cell density, e.g., E-cadherin or epithelial membrane protein 1, suppresses ferroptosis through activation of the Hippo signaling pathway 26, 27. This needs further investigations for improving the therapeutic success of ferroptosis induction in resistant epithelial cancers. HNC is commonly treated with the multimodal approach of surgery, radiotherapy, and chemotherapy 28. Systemic chemotherapy becomes more popularized in the treatment of HNC for organ-preserving strategy in combination with radiotherapy 29-31. However, up to 50% of locally advanced HNC is recurrent or persistent regardless of the multimodal treatments, which leads to poor treatment outcomes and survival 32-34. Therefore, the development of new therapeutic agents or modalities to extirpate resistant cancers is now very urgent. The present study has suggested a new therapeutic strategy for overcoming HNC chemoresistance through promoting ferroptosis with GLRX5 inhibition.

## Conclusion

This study suggests that suppression of GLRX5 promotes ferroptosis from ferroptosis inducers by increasing intracellular free iron and lipid peroxidation. GLRX5 silencing actives iron-starvation response, accountable for boosting up intracellular free iron *in vitro* and *in vivo* which in turn results in Fenton reaction and ferroptosis. The enhanced ferroptosis was rescued by the GLRX5res but not mutant GLRX5res K101Q. Therefore, our data suggested that GLRX5 inhibition predisposes therapy-resistant cancer cells to ferroptosis.

## Supplementary Material

Supplementary figures.

## References

[1] Torti SV, Manz DH, Paul BT, Blanchette-Farra N, Torti FM (2018). Iron and Cancer. Annu Rev Nutr.

[2] El Hout M, Dos Santos L, Hamai A, Mehrpour M (2018). A promising new approach to cancer therapy: Targeting iron metabolism in cancer stem cells. Semin Cancer Biol.

[3] Stockwell BR, Friedmann Angeli JP, Bayir H, Bush AI, Conrad M, Dixon SJ (2017). Ferroptosis: A regulated cell death nexus linking metabolism, redox biology, and disease. Cell.

[4] Viswanathan VS, Ryan MJ, Dhruv HD, Gill S, Eichhoff OM, Seashore-Ludlow B (2017). Dependency of a therapy-resistant state of cancer cells on a lipid peroxidase pathway. Nature.

[5] Hangauer MJ, Viswanathan VS, Ryan MJ, Bole D, Eaton JK, Matov A (2017). Drug-tolerant persister cancer cells are vulnerable to GPX4 inhibition. Nature.

[6] Yuan H, Li X, Zhang X, Kang R, Tang D (2016). CISD1 inhibits ferroptosis by protection against mitochondrial lipid peroxidation. Biochem Biophys Res Commun.

[7] Kim EH, Shin D, Lee J, Jung AR, Roh JL (2018). CISD2 inhibition overcomes resistance to sulfasalazine-induced ferroptotic cell death in head and neck cancer. Cancer Lett.

[8] Alvarez SW, Sviderskiy VO, Terzi EM, Papagiannakopoulos T, Moreira AL, Adams S (2017). NFS1 undergoes positive selection in lung tumours and protects cells from ferroptosis. Nature.

[9] Ciofi-Baffoni S, Nasta V, Banci L (2018). Protein networks in the maturation of human iron-sulfur proteins. Metallomics.

[10] Ye H, Jeong SY, Ghosh MC, Kovtunovych G, Silvestri L, Ortillo D (2010). Glutaredoxin 5 deficiency causes sideroblastic anemia by specifically impairing heme biosynthesis and depleting cytosolic iron in human erythroblasts. J Clin Invest.

[11] Liu G, Wang Y, Anderson GJ, Camaschella C, Chang Y, Nie G (2016). Functional Analysis of GLRX5 Mutants Reveals Distinct Functionalities of GLRX5 Protein. J Cell Biochem.

[12] Kim SY, Chu KC, Lee HR, Lee KS, Carey TE (1997). Establishment and characterization of nine new head and neck cancer cell lines. Acta Oto-laryngol.

[13] Roh JL, Park JY, Kim EH, Jang HJ, Kwon M (2016). Activation of mitochondrial oxidation by PDK2 inhibition reverses cisplatin resistance in head and neck cancer. Cancer Lett.

[14] Nakamura M, Nakatani K, Uzawa K, Ono K, Uesugi H, Ogawara K (2005). Establishment and characterization of a cisplatin-resistant oral squamous cell carcinoma cell line, H-1R. Oncol Rep.

[15] Ishimoto T, Nagano O, Yae T, Tamada M, Motohara T, Oshima H (2011). CD44 variant regulates redox status in cancer cells by stabilizing the xCT subunit of system xc(-) and thereby promotes tumor growth. Cancer cell.

[16] Xie Y, Hou W, Song X, Yu Y, Huang J, Sun X (2016). Ferroptosis: process and function. Cell Death Differ.

[17] Camaschella C, Campanella A, De Falco L, Boschetto L, Merlini R, Silvestri L (2007). The human counterpart of zebrafish shiraz shows sideroblastic-like microcytic anemia and iron overload. Blood.

[18] Lane DJ, Merlot AM, Huang ML, Bae DH, Jansson PJ, Sahni S (2015). Cellular iron uptake, trafficking and metabolism: Key molecules and mechanisms and their roles in disease. Biochim Biophys Acta.

[19] Bogdan AR, Miyazawa M, Hashimoto K, Tsuji Y (2016). Regulators of Iron Homeostasis: New Players in Metabolism, Cell Death, and Disease. Trends Biochem Sci.

[20] Anderson CP, Shen M, Eisenstein RS, Leibold EA (2012). Mammalian iron metabolism and its control by iron regulatory proteins. Biochim Biophys Acta.

[21] Seiler A, Schneider M, Forster H, Roth S, Wirth EK, Culmsee C (2008). Glutathione peroxidase 4 senses and translates oxidative stress into 12/15-lipoxygenase dependent- and AIF-mediated cell death. Cell Metabol.

[22] Hirschhorn T, Stockwell BR (2019). The development of the concept of ferroptosis. Free Rad Biol Med.

[23] Hassannia B, Vandenabeele P, Vanden Berghe T (2019). Targeting Ferroptosis to Iron Out Cancer. Cancer Cell.

[24] Pantopoulos K, Porwal SK, Tartakoff A, Devireddy L (2012). Mechanisms of mammalian iron homeostasis. Biochem.

[25] Biederbick A, Stehling O, Rosser R, Niggemeyer B, Nakai Y, Elsasser HP (2006). Role of human mitochondrial Nfs1 in cytosolic iron-sulfur protein biogenesis and iron regulation. Mol Cell Biol.

[26] Wu J, Minikes AM, Gao M, Bian H, Li Y, Stockwell BR (2019). Intercellular interaction dictates cancer cell ferroptosis via NF2-YAP signalling. Nature.

[27] Yang WH, Ding CC, Sun T, Rupprecht G, Lin CC, Hsu D (2019). The Hippo Pathway Effector TAZ Regulates Ferroptosis in Renal Cell Carcinoma. Cell Rep.

[28] Joo YH, Cho JK, Koo BS, Kwon M, Kwon SK, Kwon SY (2019). Guidelines for the surgical management of oral cancer: Korean Society of Thyroid-Head and Neck Surgery. Clin Exp Otorhinolaryngol.

[29] Haddad RI, Shin DM (2008). Recent advances in head and neck cancer. New Engl J Med.

[30] Choong N, Vokes E (2008). Expanding role of the medical oncologist in the management of head and neck cancer. CA Cancer J Clin.

[31] Denaro N, Merlano MC (2018). Immunotherapy in head and neck squamous cell cancer. Clin Exp Otorhinolaryngol.

[32] Argiris A, Karamouzis MV, Raben D, Ferris RL (2008). Head and neck cancer. Lancet.

[33] Argiris A, Harrington KJ, Tahara M, Schulten J, Chomette P, Ferreira Castro A (2017). Evidence-based treatment options in recurrent and/or metastatic squamous cell carcinoma of the head and neck. Front Oncol.

[34] Kim BH, Park SJ, Jeong WJ, Ahn SH (2018). Comparison of treatment outcomes for T3 glottic squamous cell carcinoma: A meta-analysis. Clin Exp Otorhinolaryngol.

