# Supplementary material for: Inhibition of Glutaredoxin 5 predisposes Cisplatin-resistant Head and Neck Cancer Cells to Ferroptosis
Source: Theranostics. 2020 Jun 19;10(17):7775–86. doi: 10.7150/thno.46903 (PMC7359084; doi:10.7150/thno.46903)
Supplement: Supplementary file 1 — Supplementary figures. [file thnov10p7775s1.pdf]

Supplementary Figures

Figure S1. Genetic inhibition of GLRX5 increases the sensitivity to ferroptosis induced by cyst(e)ine deprivation in HNC cells. (***A–B***). HN4 (cisplatin-sensitive) and HN4R (cisplatin-resistant) HNC cell lines were transfected with shGLRX5 or vector. The cells were incubated in cyst(e)ine deprivation media (CC (–)) for 48 h and then, stained using propidium iodide (PI). PI-positive cells were counted by fluorescent microscopy and quantified by the Image J software. NT indicates control not treated with cyst(e)ine deprivation. Scale bar, 50 μm. *** $P < 0.001$. (***C–D***) The levels of cellular total and lipid ROS in HNC cells were measured using DCFDA and BODIPY, respectively. The graphs showed the percentages of cells producing cellular total or lipid ROS. *** $P < 0.001$. (***E***) Cellular glutathione (GSH) levels were measured and compensated by control. The error bars represent standard errors from three replicates. NS indicates statistically not significant.

Figure S2. Resistant GLRX5 cDNA rescues HNC cells from ferroptosis. (***A–B***) Cellular iron levels in cisplatin-resistant HN3R and HN4R cells were measured by labile iron pool (LIP) assay. Calcein AM-positive cells were counted using fluorescent microscopy after cyst(e)ine deprivation (CC (–)) for 8 h and then, were quantified by the Image J software. Scale bar, 50 μm. *** $P < 0.001$. (***C***) Mitochondria iron contents were measured using rhodamine B-[(1,10-phenanthroline-5-yl)-aminocarbonyl]benzyl ester (RPA) and were compared among HN4 cells with vector, shGLRX5, and shGLRX5 plus GLRX5 cDNA (GLRX5res) after cyst(e)ine deprivation for 8 h or not. ** $P < 0.01$, *** $P < 0.001$ relative to vtr. (***D–E***) Cellular and lipid ROS were measured using flow cytometer after treatment with cyst(e)ine deprivation for 8 h. Cellular total and lipid ROS levels were measured using DCFDA and BODIPY, respectively. *** $P < 0.001$. (***F***) Cell death assay was performed using PI and then, counted using fluorescent microscopy followed by quantification using the Image J software. *** $P < 0.001$. (***G***) The amount of cellular iron was measured using LIP assay. ** $P < 0.01$, *** $P < 0.001$ relative to NT or vtr.

Figure S3. *GLRX5* mRNA expression level in and survival of the HNC cohort of the TCGA datasets. (***A***) Comparison of *GLRX5* mRNA levels between 43 normal mucosa samples and 519 HNC samples.

The bar values indicated medians. The *t*-test was used to compare *GLRX5* mRNA levels between normal and HNC samples. (***B***–***C***) Kaplan–Meier curves estimating overall survival (OS) and disease-free survival (DFS) according to low and high expression levels of *GLRX5* mRNA in the HNC patient cohort of the TCGA datasets. The log-rank test was used to compare the survival rates between groups.

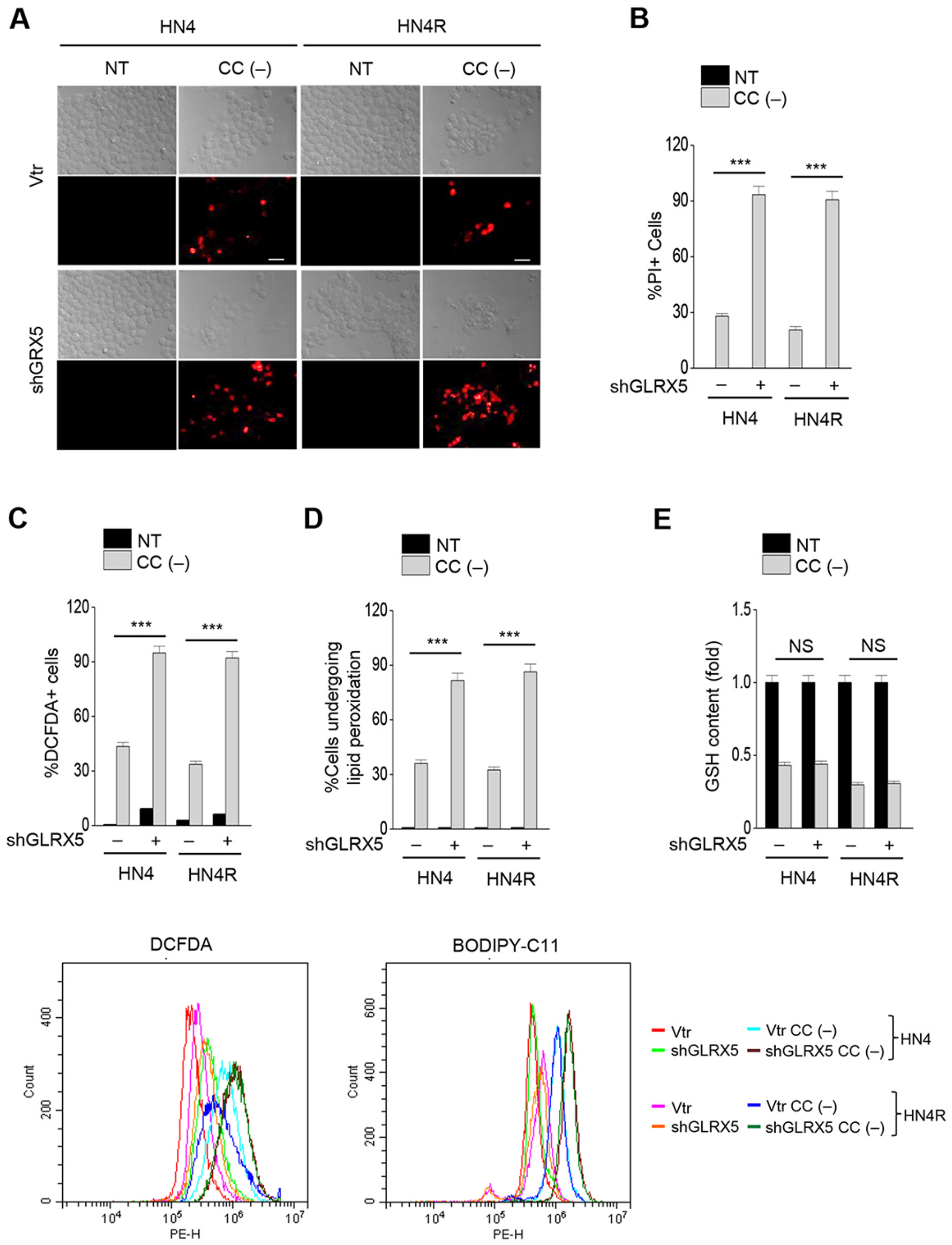

Figure S1. Genetic inhibition of GLRX5 increases the sensitivity to ferroptosis induced by cyst(e)ine deprivation in HNC cells. (***A*–*B***). HN4 (cisplatin-sensitive) and HN4R (cisplatin-resistant) HNC cell lines were transfected with shGLRX5 or vector. The cells were incubated in cyst(e)ine deprivation media (CC (–)) for 48 h and then, stained using propidium iodide (PI). PI-positive cells were counted by fluorescent microscopy and quantified by the Image J software. NT indicates control not treated with cyst(e)ine deprivation. Scale bar, 50 µm. *** $P < 0.001$. (***C*–*D***) The levels of cellular total and lipid ROS in HNC cells were measured using DCFDA and BODIPY, respectively. The graphs showed the percentages of cells producing cellular total or lipid ROS. *** $P < 0.001$. (***E***) Cellular glutathione (GSH) levels were measured and compensated by control. The error bars represent standard errors from three replicates. NS indicates statistically not significant.

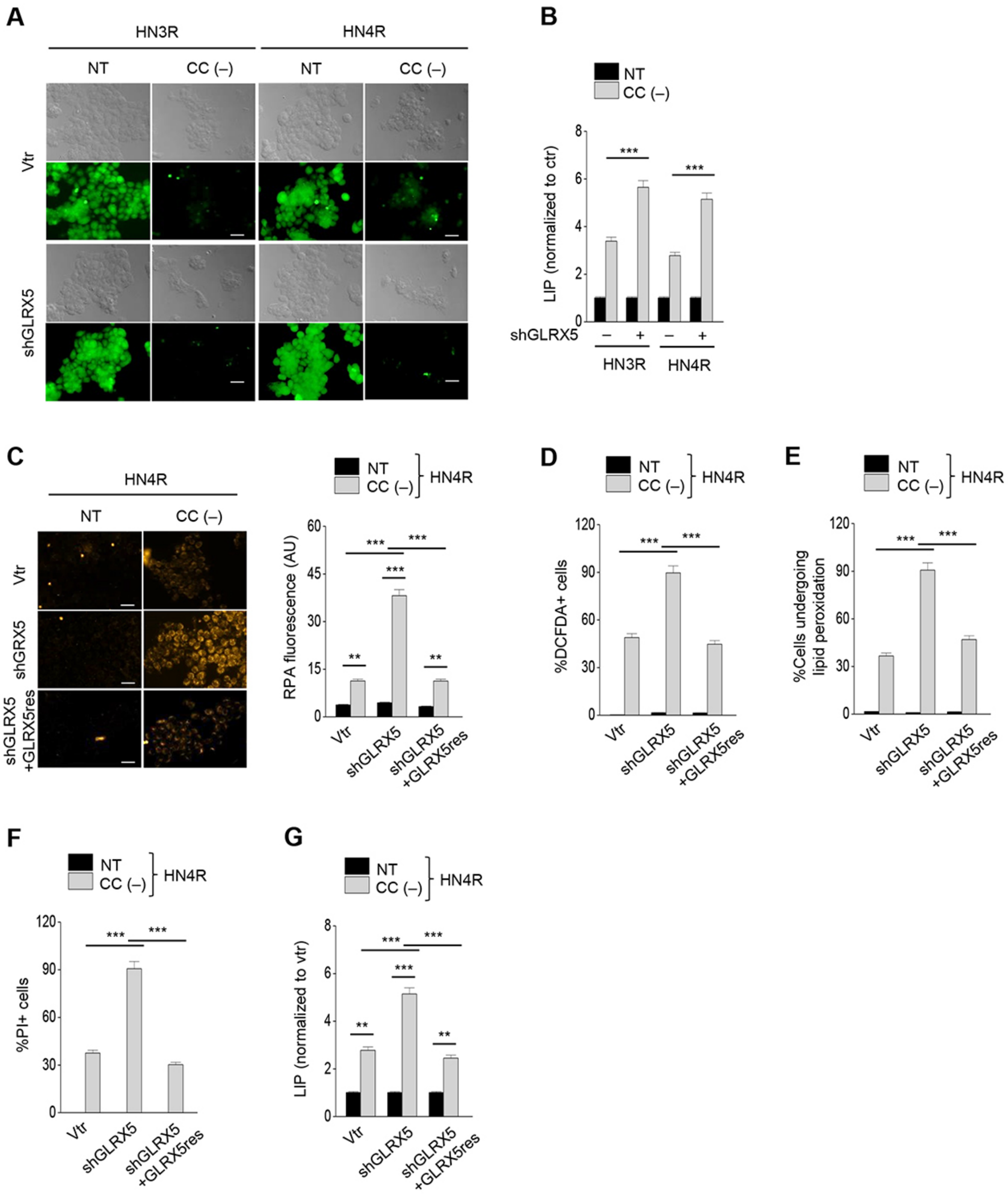

Figure S2. Resistant GLRX5 cDNA rescues HNC cells from ferroptosis. (***A*–*B***) Cellular iron levels in cisplatin-resistant HN3R and HN4R cells were measured by labile iron pool (LIP) assay. Calcein AM-positive cells were counted using fluorescent microscopy after cyst(e)ine deprivation (CC (–)) for 8 h and then, were quantified by the Image J software. Scale bar, 50 μm. *** $P < 0.001$. (***C***) Mitochondria iron contents were measured using rhodamine B-[(1,10-phenanthroline-5-yl)-aminocarbonyl]benzyl ester (RPA) and were compared among HN4 cells with vector, shGLRX5, and shGLRX5 plus GLRX5 cDNA (GLRX5res) after cyst(e)ine deprivation for 8 h or not. ** $P < 0.01$, *** $P < 0.001$ relative to vtr. (***D*–*E***) Cellular and lipid ROS were measured using flow cytometer after treatment with cyst(e)ine deprivation for 8 h. Cellular total and lipid ROS levels were measured using DCFDA and BODIPY, respectively. *** $P < 0.001$. (***F***) Cell death assay was performed using PI and then, counted using fluorescent microscopy followed by quantification using the Image J software. *** $P < 0.001$. (***G***) The amount of cellular iron was measured using LIP assay. ** $P < 0.01$, *** $P < 0.001$ relative to NT or vtr.

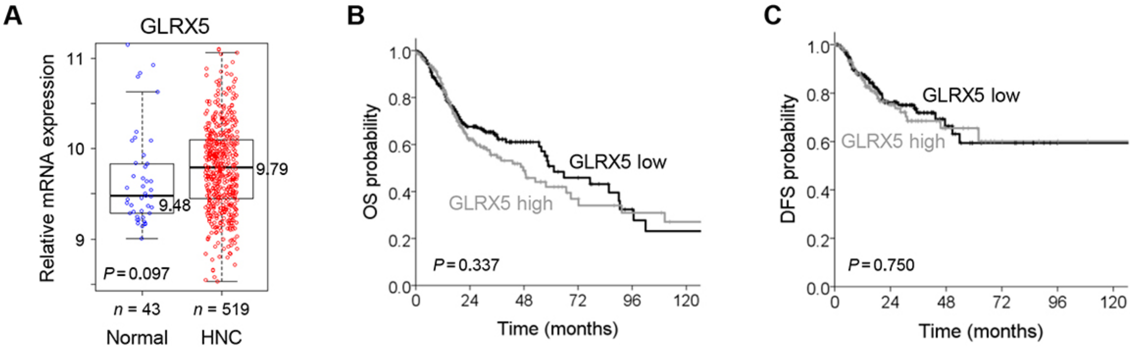

Figure S3. *GLRX5* mRNA expression level in and survival of the HNC cohort of the TCGA datasets. (***A***) Comparison of *GLRX5* mRNA levels between 43 normal mucosa samples and 519 HNC samples. The bar values indicated medians. The *t*-test was used to compare *GLRX5* mRNA levels between normal and HNC samples. (***B***–***C***) Kaplan–Meier curves estimating overall survival (OS) and disease-free survival (DFS) according to low and high expression levels of *GLRX5* mRNA in the HNC patient cohort of the TCGA datasets. The log-rank test was used to compare the survival rates between groups.
